# Utilization of a cell‐penetrating peptide‐adaptor for delivery of human papillomavirus protein E2 into cervical cancer cells to arrest cell growth and promote cell death

**DOI:** 10.1002/cnr2.1810

**Published:** 2023-03-28

**Authors:** Julia C. LeCher, Hope L. Didier, Robert L. Dickson, Lauren R. Slaughter, Juana C. Bejarano, Steven Ho, Scott J. Nowak, Carol A. Chrestensen, Jonathan L. McMurry

**Affiliations:** ^1^ Center for ViroScience and Cure, Laboratory of Biochemical Pharmacology, Department of Pediatrics Emory University School of Medicine Atlanta Georgia 30322 USA; ^2^ Department of Molecular & Cellular Biology Kennesaw State University 370 Paulding Ave NW, MD 1201 Kennesaw Georgia 30144 USA; ^3^ Department of Chemistry & Biochemistry Kennesaw State University 370 Paulding Ave NW, MD 1203 Kennesaw Georgia 30144 USA

**Keywords:** cell‐penetrating peptides, cervical cancer, E2, E6, E7, HPV‐16

## Abstract

**Background:**

Human papillomavirus (HPV) is the causative agent of nearly all forms of cervical cancer, which can arise upon viral integration into the host genome and concurrent loss of viral regulatory gene E2. Gene‐based delivery approaches show that E2 reintroduction reduces proliferative capacity and promotes apoptosis in vitro.

**Aims:**

This work explored if our calcium‐dependent protein‐based delivery system, TAT‐CaM, could deliver functional E2 protein directly into cervical cancer cells to limit proliferative capacity and induce cell death.

**Materials and Results:**

TAT‐CaM and the HPV16 E2 protein containing a CaM‐binding sequence (CBS‐E2) were expressed and purified from *Escherichia coli*. Calcium‐dependent binding kinetics were verified by biolayer interferometry. Equimolar TAT‐CaM:CBS‐E2 constructs were delivered into the HPV16^+^ SiHa cell line and uptake verified by confocal microscopy. Proliferative capacity was measured by MTS assay and cell death was measured by release of lactate dehydrogenase. As a control, human microvascular cells (HMECs) were used. As expected, TAT‐CaM bound CBS‐E2 with high affinity in the presence of calcium and rapidly disassociated upon its removal. After introduction by TAT‐CaM, fluorescently labeled CBS‐E2 was detected in cellular interiors by orthogonal projections taken at the depth of the nucleus. In dividing cells, E2 relocalized to regions associated with the mitotic spindle. Cells receiving a daily dose of CBS‐E2 for 4 days showed a significant reduction in metabolic activity at low doses and increased cell death at high doses compared to controls. This phenotype was retained for 7 days with no further treatments. When subcultured on day 12, treated cells regained their proliferative capacity.

**Conclusions:**

Using the TAT‐CaM platform, bioactive E2 protein was delivered into living cervical cancer cells, inducing senescence and cell death in a time‐ and dose‐dependent manner. These results suggest that this nucleic acid and virus‐free delivery method could be harnessed to develop novel, effective protein therapeutics.

## 1 INTRODUCTION

Human papillomavirus is a sexually transmitted virus and the causative agent of multiple forms of cancer including cervical, vaginal, oropharyngeal, anal, penile and vulvar and is the second leading cause of cancer‐related death in women worldwide.^1^ Globally, this is partly attributed to a lack of access to preventive care and early detection, particularly in middle and low‐income nations. Further, metastatic cervical cancer remains difficult to treat and retains high 5‐year recurrence rates. Recent years have seen a surge in clinical trials aimed at developing new immunotherapies to increase survival rates but HPV‐mediated cervical cancer still remains a significant global burden and new treatment approaches are wanting.

A key event in many HPV‐mediated cancers is viral integration into the human genome. During primary infection, HPV infects undifferentiated cells of the cervical basal epithelium. New virions exit from terminally differentiated cells in the outer layer of the cervical epithelium. The virus thus requires proliferation and subsequent differentiation of host endodermal cells up the cervical epithelial wall for egress of new virions.^2^ To insure this occurs, HPV encodes two proteins, E6 and E7, that inhibit apoptotic pathways and promote cellular proliferation, respectively.^3^, ^4^ Another viral protein, E2, regulates E6 and E7 at the level of transcription and via direct protein binding.^5^, ^6^, ^7^ In over 80% of HPV carcinomas the E2 open reading frame (ORF) is the primary site of viral integration. Integration often results in the loss of E2 but retention of the E6 and E7 ORFs.^8^, ^9^, ^10^, ^11^, ^12^ This promotes unregulated overproduction of E6 and E7 which, in turn, can lead to cellular changes promoting carcinogenesis. Loss of E2 is thought to be a critical event in the onset of many integrated HPV cancers.

Given its regulatory role of inhibiting E6 and E7, in 1993 Hwang et al. hypothesized that replenishment of E2 in cervical cancer cells could halt their proliferation and reverse their metastatic potential.^13^ They, and others, demonstrated that reintroduction of E2 into cervical cancer cells could induce cell senescence.^13^, ^14^, ^15^ Later work showed that E2 overexpression after gene delivery promotes apoptosis.^16^, ^17^ However promising, this approach has not become a viable treatment option for cancer patients likely owing to the need for gene transfection, a technical challenge in and of itself.^18^ In 2004, Roeder et al. described the use of the HSV cell‐penetrating peptide (CPP), VP22, to deliver VP22:E2 fusion proteins into cervical cancer cell lines for the induction of apoptosis.^19^ In this, and later studies, VP22:E2 fusion proteins were made from plasmids introduced into cells and, once translated, these fusion proteins were secreted from transformed cells and readily entered other neighboring cells to promote cell death.^19^, ^20^ In this study we developed a more direct approach for E2 protein delivery using a CPP TAT‐CaM adaptor.

CPPs are so named because they were originally thought to readily cross cell membranes, carrying with them molecular “cargo” molecules to which they are attached. However, current thought is that most CPPs directly bind to plasma membrane receptors and enter cells via endocytosis. Endocytic entry most commonly results in the degradation of the membrane‐bound CPP instead of release into the cytoplasm. Irreversible attachment of CPP to cargo, most commonly via covalent bond or nonspecific hydrophobic interaction, results in the cargo becoming trapped in the endosome and targeted for degradation along with the CPP.^21^ Our CPP adaptor, “TAT‐CaM,” consists of well‐known CPP derived from HIV transactivator of transcription (TAT), fused to a human calmodulin (CaM).^22^, ^23^ Cargo proteins are engineered to contain a calmodulin binding sequence (CBS). TAT‐CaM‐mediated delivery relies on the relatively high levels of calcium in the extracellular milieu in which TAT‐CaM:cargo complexes spontaneously bind with high affinity and remain tightly associated upon entry into the cell. However, during endosomal trafficking, calcium efflux from the endosome allows for cargo dissociation from TAT‐CaM. After disassociation, the cargo is free and subsequently released to the cytoplasm of living mammalian cells while TAT‐CaM remains bound to the endosome. Delivery is rapid, tunable, and efficient and a wide variety of cargos can be delivered into living cells.^22^, ^23^, ^24^, ^25^

Using the TAT‐CaM system, we tested the hypothesis that TAT‐CaM could deliver free, bioactive CBS‐E2 directly into cervical cancer cells to inhibit cellular proliferation and/or cell death. Following delivery, CBS‐E2 showed distinct cell‐cycle dependent subcellular localization patterns and was found in both the cytoplasm and the nucleus. In mitotic cells, CBS‐E2 relocalized to regions of the cell associated with the mitotic spindle, a known biological activity.^26^ As expected, CBS‐E2 prohibited cellular proliferation and promoted cell death in a time and dose‐dependent manner supporting a model wherein E2 reduces cellular proliferation at low cell‐to‐peptide ratios and promotes cell death at high cell‐to‐peptide ratios. These data also further validate the TAT‐CaM adaptor and provide a new framework for delivery of E2 protein into living cells.

## 2 MATERIALS AND METHODS

### 2.1 Generation and purification of CBS‐E2 and TAT‐CaM constructs

An Escherichia coli (*E. coli*)‐optimized synthetic gene encoding the E2 ORF from HPV‐16 was cloned into pCAL‐N‐FLAG (Agilent Technologies) containing an N‐terminal calmodulin bind site (CBS). CBS‐E2 and TAT‐CaM were expressed and purified as previously described with slight modifications.^22^ Briefly, CBS‐E2 was expressed in ArcticExpress (DE3) *E. coli* cells (Agilent Technologies) and purified by fast protein liquid chromatography using Calmodulin‐Sepharose (GE Healthcare). TAT‐CaM was expressed in BL21(DE3)pLysS *E. coli* cells (Agilent Technologies) and purified to near‐homogeneity by metal‐affinity chromatography using TALON resin (Takara Bio). After purification, protein constructs were dialyzed into calcium‐containing binding buffer (10 mM HEPES, 150 mM NaCl, 2 mM CaCl_2_, 10% glycerol pH 7.4), sterilized via syringe‐driven filtration through a 0.22 μm filter, flash frozen in liquid nitrogen and stored at −80°C until use. Samples were collected at each stage of the purification process in 2% SDS buffer and subjected to gel electrophoresis as previously described.^27^ Elutions were further subjected to immunoblot analysis as previously described using an HPV 16 E2 monoclonal primary antibody TVG 621 (ThermoFisher) and goat anti‐mouse HRP conjugated secondary (ThermoFisher).^27^

### 2.2 Biolayer Interferometry

Biolayer interferometry (BLI) experiments were performed on a FortéBio Octet QK (Menlo Park) as previously described.^22^ Biotinylated TAT‐CaM was loaded onto streptavidin (SA) sensors for 300 s in binding buffer followed by a 180 s baseline measurement. TAT‐CaM ligand was then exposed to analyte CBS‐E2 and association was measured for 300 s. Two different dissociation phases followed, each 300 s in length. Ligand: analyte pairs were first exposed to binding buffer and were then challenged in binding buffer containing 10 mM EDTA. Signal resulting from baseline drift was measured by a parallel run in which a ligand‐loaded sensor was exposed to buffer only. This was subtracted from each experimental run prior to analysis. Fast‐on, slow‐off binding was fit to a global 1:1 association‐then‐dissociation model and EDTA‐induced rapid dissociation was separately fit to a one‐phase exponential decay model using GraphPad Prism 5.02 software. Nonspecific binding, as measured by a run of a sensor without ligand exposed to the highest concentration of CBS‐E2, evinced negligible binding and was ignored in analysis.

### 2.3 Cell culture

The HPV‐16+ cell line SiHa (ATCC© HTB‐35) and the Human Microvascular Endothelial Cell line (HMEC; CRL‐3243) were purchased from ATCC (Manassas). SiHas were cultured in glucose‐free complete Dulbecco's Minimal Eagle Media (DMEM; Gibco™ ThermoFisher) with 10% fetal bovine serum (FBS; Atlas Biologicals) and 1 mM L‐glutamine (Gibco™ ThermoFisher). HMECs were cultured in MCDB131 media containing 10% FBS, 10 mM L‐Glutamine, 10 ng/mL human recombinant epidermal growth factor (EGF; Gibco™ ThermoFisher), and 1 μg/mL hydrocortisone (Gibco™ ThermoFisher). Both cell lines were maintained in a humidified incubator at 37°C with 5% CO_2_ injection.

### 2.4 Confocal microscopy

All confocal experiments were performed on an inverted Zeiss LSM700 confocal microscope equipped with a humidified incubator at 37°C with 5% CO_2_ injection as previously described.^22^ In short, SiHa cells were plated at ~50% confluency in 4‐well Nunc Lab‐Tek chambered coverglass wells (ThermoFisher) 16 h prior to cell penetration assays. CBS‐E2 cargos were labeled with DyLight 550 (ThermoFisher) or left unlabeled (experimental control) then incubated with or without (experimental control) TAT‐CaM in equimolar amounts (1 μM) in binding buffer. Complexes were then added to glucose‐free DMEM and introduced to cells. Uptake was performed in a humidified incubator at 37°C with 5% CO_2_ injection for 1 h with periodic rocking (every 15 min) to ensure even distribution. After 1 h, media were removed and cells washed five times with calcium‐containing phosphate buffered saline (PBS; 1 mM CaCl_2_). Next, cells were counterstained with 2 μM CellTracker Green CMFDA dye (ThermoFisher) and 3 μM NucBlue (ThermoFisher) per manufacturer's protocols to stain the cytoplasmic and nuclear compartments of the cells respectively. After staining, cells were washed three times with PBS + 1 mM CaCl_2_ then fresh cell culture media was added to each well. For live‐cell uptake with downstream immunofluorescence, after treatment cells were counterstained with NucBlue only then fixed in ice‐cold 100% methanol for 3 min. Fixed cells were blocked (PBS + 2% FBS), incubated overnight with primary antibody beta‐tubulin in PBS + 0.1% Triton‐X‐100, washed three times with PBS, incubated for 1 h with GFP‐conjugated secondary antibody, washed three times with PBS, mounted and visualized. Cells were imaged using a ×40 EC Plan‐Neofluar objective with a NA value of 1.3. Image analysis was performed on Zen Blue software (Carl Zeiss Microscopy) as previously described.^22^

### 2.5 Analysis of cellular proliferation and cell death

Cellular proliferation was assayed by ability to metabolize 3‐(4,5‐dimethylthiazol‐2‐yl)‐5‐(3‐carboxymethoxyphenyl)‐2‐(4‐sulfophenyl)‐2H‐tetrazolium (MTS; CellTiter 96® AQ_ueous_ One Solution Cell Proliferation Assay by Promega). In the same population of cells, cell death was assayed by release of lactate dehydrogenase (LDH) into cell culture media (CytoTox 96® Non‐Radioactive Cytotoxicity Assay by Promega). On Day 0, cells (2.5 × 10^3^ or 2.5 × 10^4^ cells per well) were seeded into 96‐well plates in 100 μL of phenol‐red free cell culture media and allowed to adhere to the plate overnight. The next day (Day 1) cells were treated with increasing concentrations of CBS‐E2 and equimolar TAT‐CaM in binding buffer (experimental group), TAT‐CaM‐alone (vehicle control), buffer (experimental control) or left untreated (untreated control). After 1 h, treatments were removed and 100 μL of cell culture medium was added to each well. Treatments were repeated at 24 and 48 h. Every 24 h, 50 μL of medium was transferred to another 96 well plate and assayed for LDH per manufacturer's protocol. At 72 h (Day 4), MTS reagent was added directly to cells and cells were assayed for MTS metabolism per manufacturer's protocol. A BioTek multimode plate reader (BioTek Instruments) was used to measure OD_490_. Absorbance due to metabolic or LDH activity was calculated by subtracting background (cells with no reagent) from total. Percent metabolic activity (MTS assay) was calculated using the following equation: (OD_treated_/OD_untreated_) × 100. Percent cell death (LDH assay) calculated using the following equation: (OD_untreated_/OD_treated_) × 100. MTS and LDH assays were run in triplicate with three technical replicates (*n* = 9).

### 2.6 Statistical analysis

All analysis was performed on GraphPad Prism 8.0 software. Average values from two or three technical replicates (as indicated in the figure legends) were obtained per experiment and average values from three biological replicates were compared. Analysis was performed by one‐way or two‐way ANOVA with either Tukey's or Dunnet's correction for multiple comparisons as indicated in figure legends. In all cases, the multiple comparisons that were performed were between (1) untreated versus vehicle‐treated, (2) untreated versus treated, and (3) vehicle‐treated versus treated to ensure any observed effect was from the treatment alone. Pairwise comparisons are given on individual graphs. Deviation was calculated using standard error of the mean.

## 3 RESULTS

### 3.1 CBS‐E2 binds TAT‐CaM with expected kinetics

**FIGURE 1:**
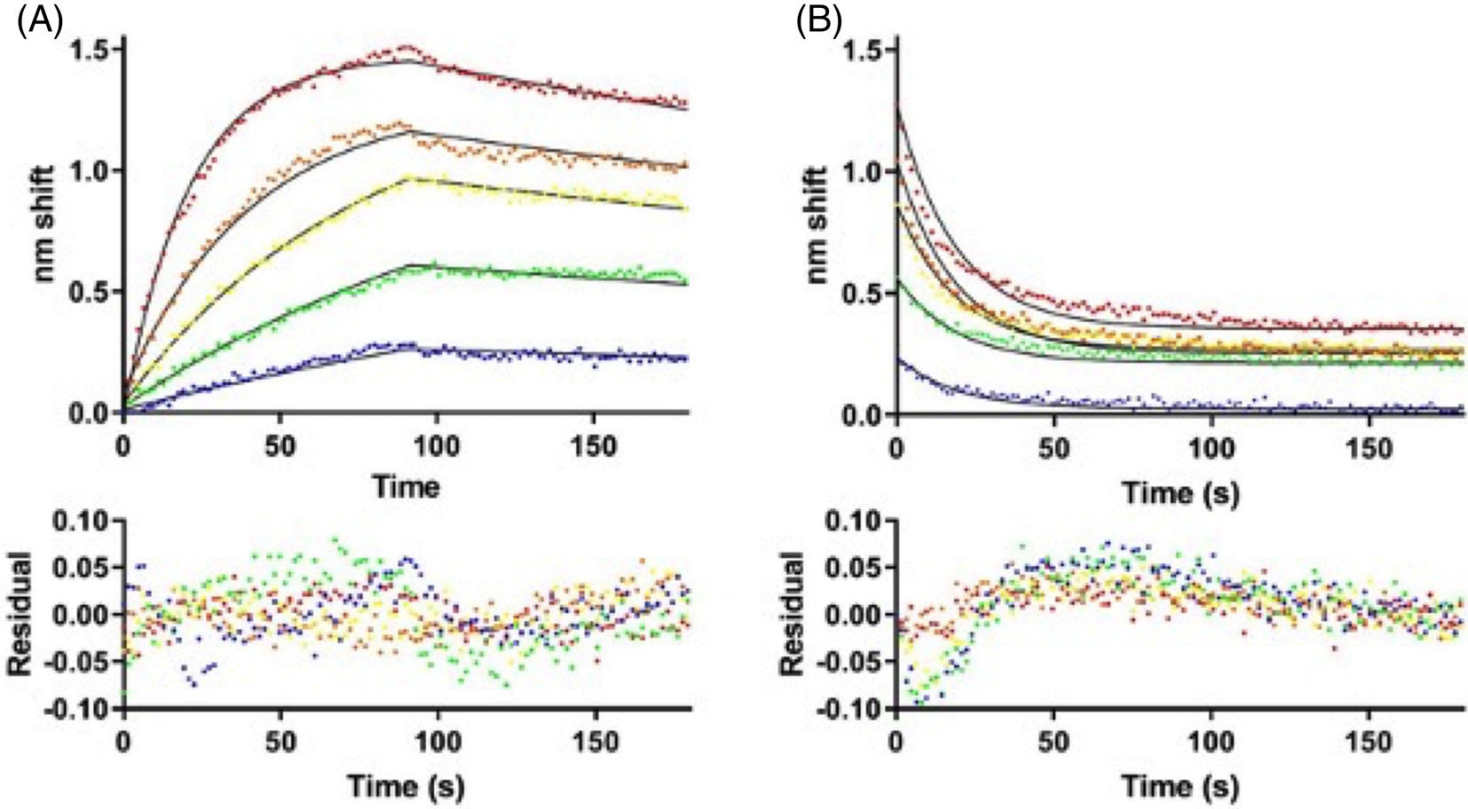
Biolayer interferometry analysis of TAT‐CaM binding to CBS‐E2. (A) Association‐then‐dissociation experiment in which ligand TAT‐CaM was exposed to varying concentrations of CBS‐E2 prior to movement to buffer only at 90s (red, 1000 nM; orange, 500 nM; yellow, 250 nM, green, 125 nM, blue 63 nM). Data points are individual instrument readings. Lines represent best fits to a global single‐state model. Residuals are shown below. (B) The same samples after dissociation were moved to buffer containing 10 mM EDTA for monitoring of dissociation in the absence of Ca^2+^. Fits are to a global single‐state exponential decay model. Residuals indicate some non‐ideality in the model, likely due to rapid dissociation prior to the first reading (see discussion).

Our previous work validated that TAT‐CaM binds CBS‐cargo proteins rapidly and stably in the presence of calcium and dissociates almost instantaneously and completely when calcium is removed.^22^, ^23^ In this study, we expressed and purified an E2 construct from HPV‐16 that contained an N′‐terminal CBS tag (Figure S1). Calcium‐dependent binding kinetics of TAT‐CaM with CBS‐E2 were analyzed via biolayer interferometry (Figure 1). Fits to a single‐state association‐then‐dissociation model (Figure 1A) yielded a calcium‐replete K_D_ of 36 nM with k_on_ = 4.6 × 10^4^ M^−1^ s^−1^ and k_off_ = 1.6 × 10^−3^ s^−1^. In the presence of the chelating agent EDTA, dissociation was very rapid (Figure 1B). k_off(EDTA)_ was 5.3 × 10^−2^ s^−1^. These data validate the utility the TAT‐CaM approach for delivery of E2.

### 3.2 Live cell uptake and cellular redistribution of CBS‐E2 post‐TAT‐CaM‐mediated delivery

**FIGURE 2:**
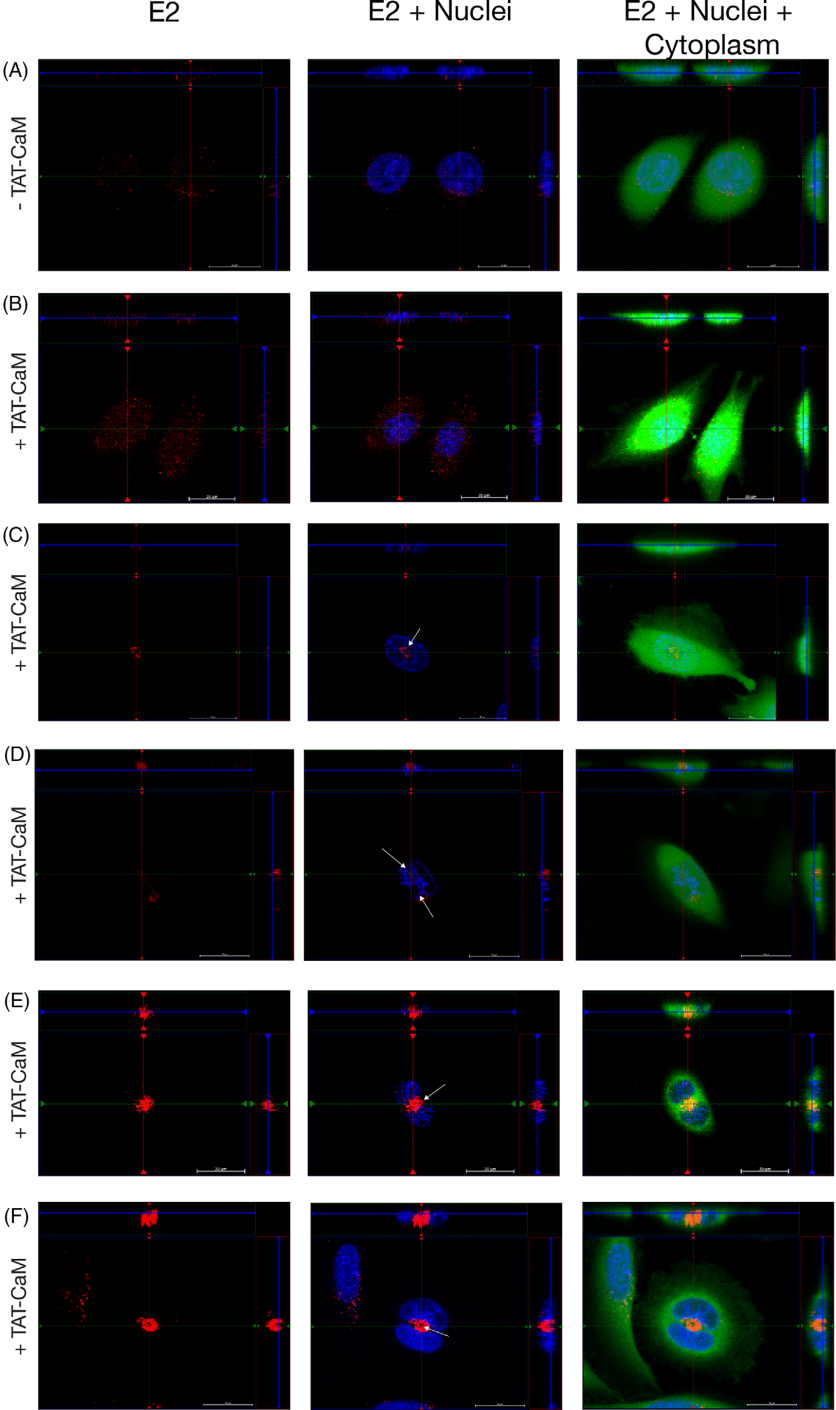
Delivery of CBS‐E2 into living cervical cancer cells via the TAT‐CaM adaptor. Cervical cancer cells were incubated with fluorescently labeled CBS‐E2 cargo (Red) in the absence (A) or presence (B–F) of equimolar TAT‐CaM for 1 h. Cells were counterstained with NucBlue (nuclei; blue) and Cytotracker (cytoplasm; green). Images were generated on an inverted Zeiss LSM700 Confocal Microscope with Z‐stack projections. Shown at the top and right of each image are orthogonal projections taken at the depth of the nucleus. (A, B) Visualization of E2 in SiHas in asynchronous populations. (C–F) Visualization of CBS‐E2 in mitotically active cells. White arrows indicate redistribution and clustering of CBS‐E2 to regions of the cell typically associated with the mitotic spindle apparatus.

TAT‐CaM was used to deliver free bioactive CBS‐tagged E2 protein into the human HPV‐16+ cervical cancer cell line SiHa. Given significant artifacts resulting from fixation that have confounded CPP results in the past, live cell imaging in asynchronous populations of human cervical cancer cells was performed. Using confocal microscopy, orthogonal projections were taken at the depth of the midpoint of the nucleus and were analyzed for intracellular delivery of fluorescently labeled CBS‐E2 (Figure 2B). To verify that TAT‐CaM mediated entry, parallel control experiments without TAT‐CaM were performed (Figure 2A). In the presence of TAT‐CaM, CBS‐E2 was readily delivered into cells (Figure 2B), while in the absence of TAT‐CaM negligible signal was observed (Figure 2A). One biological property of HPV E2 proteins is the ability to localize to the mitotic spindle during cellular division.^26^, ^28^ Circular clusters of CBS‐E2 formed on DNA were observed at the onset of mitosis (white arrows; Figure 2C,D), suggestive of localization to aster microtubules as previously described.^26^, ^28^ In cells undergoing anaphase and telophase (as determined by visual observation of nuclear staining patterns), CBS‐E2 clustered on the midplane (white arrows; Figure 2E,F). Further, live cell uptake coupled to downstream immunofluorescence showed co‐localization of CBS‐E2 with beta‐tubulin around the nucleus in cells visually undergoing mitosis (Figure S2). These data demonstrate that CBS‐E2 was delivered in bioactive form.

### 3.3 E2 inhibits cell progression and induces cell death in cervical cancer cells

**FIGURE 3:**
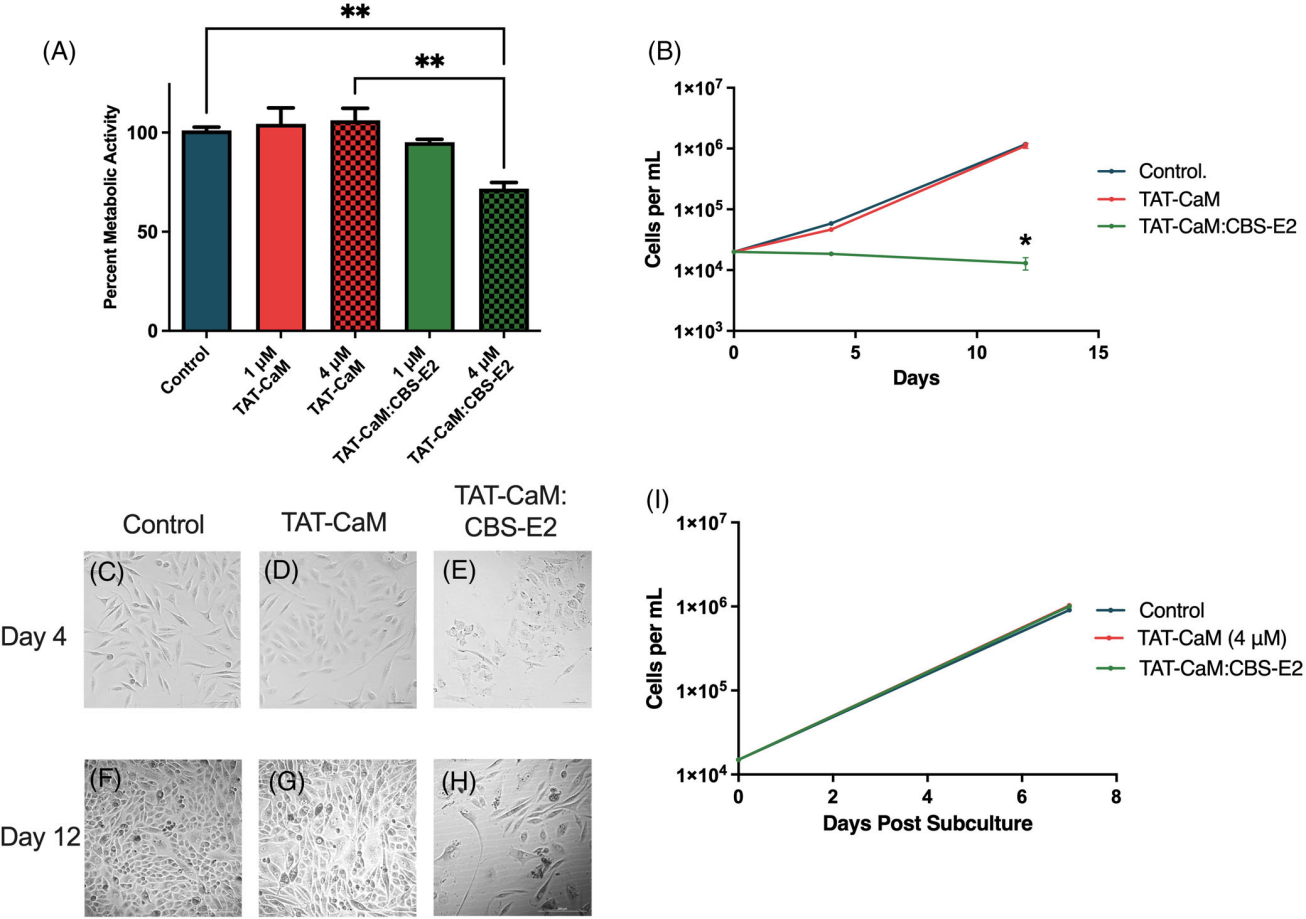
CBS‐E2 delivery induces reversible inhibition of cell growth in cervical cancer cells. Cells were seeded at 2.5 × 10^4^ per well and treated once daily for 3 days with either 1 or 4 μM CBS‐E2 in the presence of equimolar amounts of TAT‐CaM. As a control, cells were either left untreated (negative control) or treated with TAT‐CaM only (experimental control). (A) MTS assay to assess cellular metabolic activity on day 4. A reduction in metabolic activity was tested for by One‐way ANOVA with Dunnet's correction for multiple comparisons **control to CBS‐E2 4 μM *p* = .004, **TAT‐CaM 4 μM to CBS‐E2 4 μM *p* = .001. *n* = 9 for all groups; shown SEM. (B) On day 4 and day 12, cells were collected and counted on a hemocytometer. Data were analyzed by Two‐way ANOVA with Dunnet's correction for multiple comparisons to assess the effect of both time and treatment. *p*‐value indicates comparison between the TAT‐CaM and the TAT‐CaM:CBS‐E2 group *p = .02. *n* = 4 for all groups; shown SEM. (C–H) Micrographs of cells from each treatment group on day 4 and day 12 post‐treatment. Images are from the 4 μM treatments. (I) On day 12, cells were collected and reseeded at equal density and cultured for an additional week after which they were collected and counted on a hemocytometer. *n* = 4 for all groups.

Previous studies showed that transfection of cervical cancer cells with E2 is sufficient for induction of senescence or apoptosis within 3 days.^13^, ^14^, ^15^, ^16^ An experimental limitation to the use of gene delivery is lack of control of dose, that is, how much protein is made in the cell after transfection. To test if CBS‐E2 could induce senescence and/or cell death by direct protein delivery, experiments were designed to determine how much protein would be required over 3 days. As a starting point, 2.5 × 10^4^ cells were treated daily for 3 days with 1 or 4 μM doses of CBS‐E2 and equimolar TAT‐CaM. At 1 μM there was no significant effect on cells post CBS‐E2 delivery, while at 4 μM, there was a 28% reduction in metabolic activity on day 4 compared with TAT‐CaM‐alone‐treated cells (Figure 3A). Total cell counts were also performed at day 4. Untreated and TAT‐CaM‐alone‐treated groups showed similar growth rates while cells treated with 4 μM CBS‐E2 failed to proliferate (Figure 3B). Microscopic analysis of cells on day 4 further corroborated these findings (Figure 3C–E). Untreated and TAT‐CaM‐alone‐treated cells exhibited normal morphology. In contrast, CBS‐E2‐treated cells lost their spindle‐like morphology and exhibited intracellular stress granule‐like formations (Figure 3E). Collectively, these data support that three doses of CBS‐E2 protein over 3 days is sufficient to significantly reduce cellular proliferation within this population of cells.

Persistence of this phenotype was assayed by retaining cells in culture for an additional week with regular media changes. Over 12 days, with no additional CBS‐E2 treatments, cells from the 4 μM CBS‐E2 treated group failed to proliferate while untreated cells and those dosed with TAT‐CaM only retained normal doubling times (Figure 3B). Microscopic analysis supported these findings (Figure 3F–H). In untreated and TAT‐CaM‐treated groups, cells became over‐confluent and crowded the wells (Figure 3F,G). CBS‐E2‐treated cells showed no increase in cell number (Figure 3B), however, some cells within the population regained normal spindle‐like morphology (Figure 3H). Like many other cancer cell lines, the SiHa cell line is a heterogenous population with genotypic and phenotypic cellular diversity. We hypothesized that the cells in our experiment that regained normal morphology might represent a subpopulation of harder to treat cells within the population. To test for this, cells were collected and re‐seeded at equal density. After 7 days in culture, cells were collected and counted (Figure 3I). CBS‐E2‐treated cells regained normal growth kinetics (Figure 3I) and normal morphology (Figure 3B) indistinguishable from untreated or TAT‐CaM‐alone‐treated cells. These data suggest that at the cell‐to‐peptide ratios employed only a sub‐population of cells underwent senescence while others were seemingly unaffected or more resistant to CBS‐E2's effects.

### 3.4 Dose‐dependent effect of E2 on cellular proliferation and cell death

**FIGURE 4:**
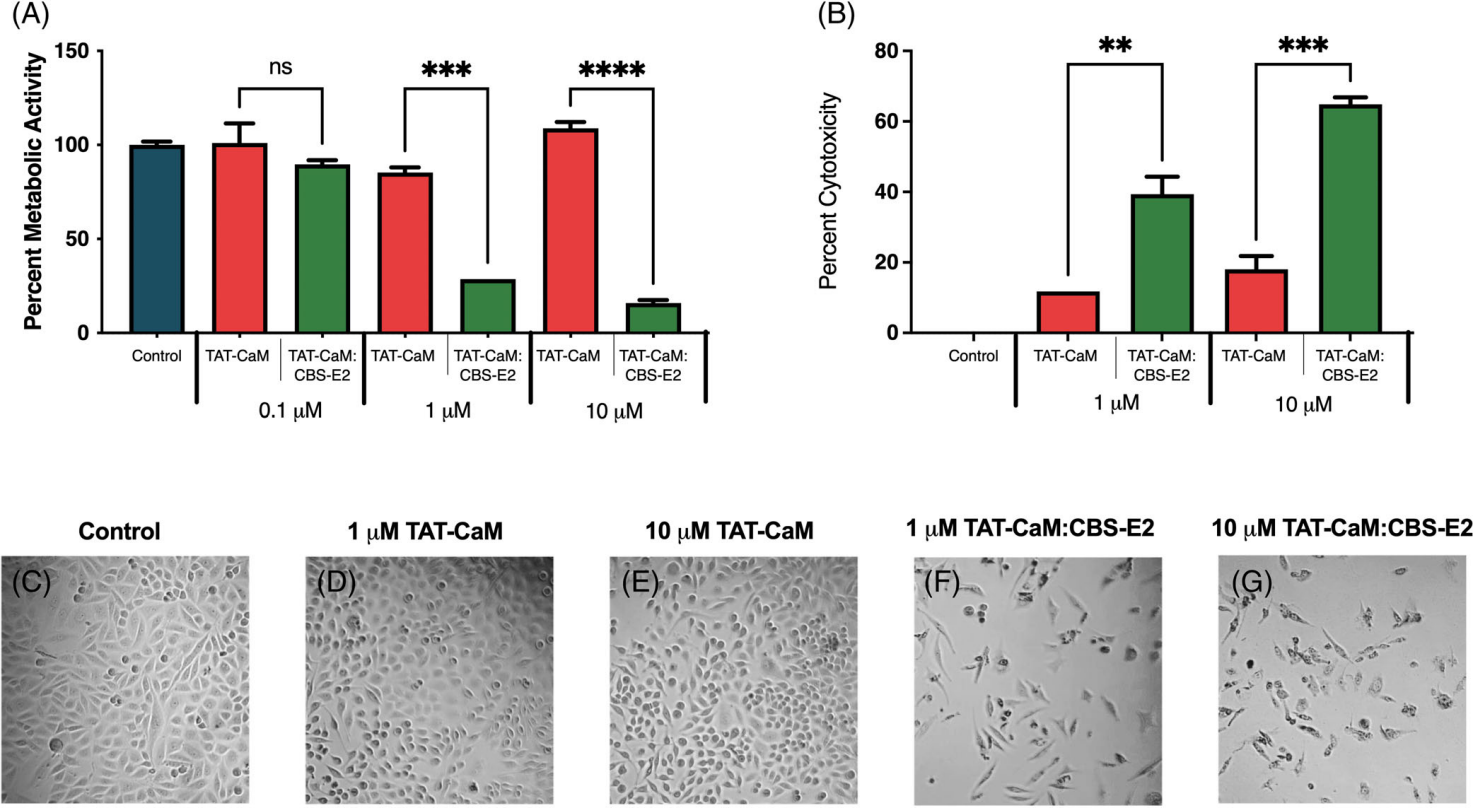
CBS‐E2 induces cell death in cervical cancer cells. Cells were seeded at 2.5 × 10^3^ and treated once daily for 3 days with either 1, 3 or 10 μM CBS‐E2 in the presence of equimolar amounts of TAT‐CaM. As a control, cells were either left untreated (negative control) or treated with TAT‐CaM only (experimental control). (A) MTS assay to assess cellular metabolic activity on day 4. A reduction in metabolic activity was tested for by One‐way ANOVA with Dunnet's correction for multiple comparisons. *p*‐value indicates comparison between the TAT‐CaM and the TAT‐CaM:CBS‐E2 group at same concentrations ****p < .001. *n* = 9 for all groups; shown SEM. (B) LDH leakage assay to assess cytotoxicity on day 4. Percent cytotoxicity was tested for by one‐way ANOVA with Dunnet's correction for multiple comparisons. *p*‐value indicates comparison between the TAT‐CaM and the TAT‐CaM:CBS‐E2 group at same concentration ***p = .007, ***p = .0006, *n* = 9 for all groups; shown SEM. (C–G) Micrographs of cells from each treatment group taken on day 4.

**FIGURE 5:**
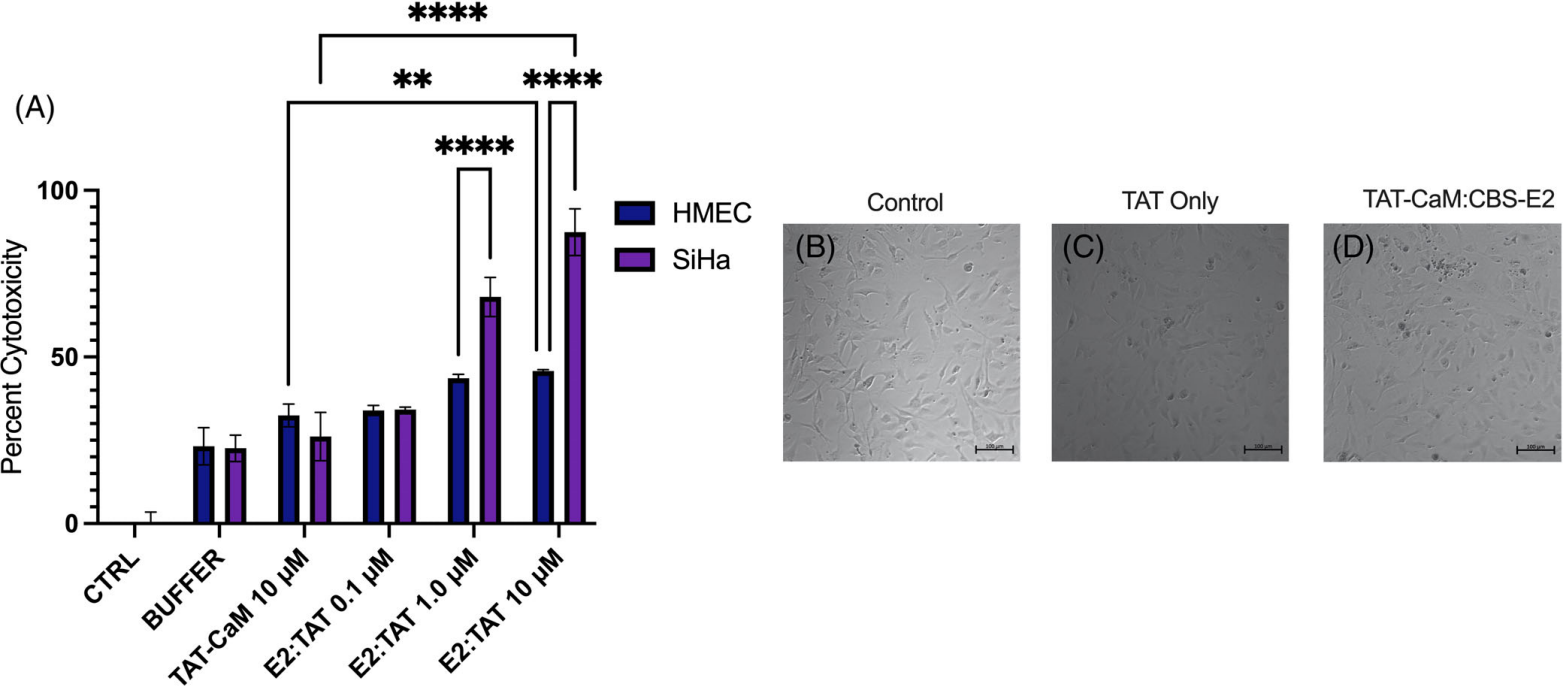
CBS‐E2 does not induce cell death in human microvascular endothelial cells. SiHa and HMEC cells were seeded at 2.5 × 10^3^ and treated once daily for 3 days with 10 μM CBS‐E2 in the presence of equimolar amounts of TAT‐CaM. As a control, cells were either left untreated (negative control), treated with NEB buffer (treatment control), or treated with TAT‐CaM only (experimental control). (A) LDH leakage assay to assess cytotoxicity on day 4. Percent cytotoxicity were tested for by One‐way ANOVA with Dunnet's correction for multiple comparisons. *n* = 4 for all groups; shown SEM. *p*‐values indicate comparisons between the two different cells types at the same concentrations ****p < 0.0001. (B–D) Micrographs of cells from each treatment group taken on day 4.

To test the effect of cell‐to‐peptide ratios, a dose–response assay was performed using the same protocol with the exception that the starting cell number was lowered 10‐fold. While 0.1 μM doses had little effect, cells showed a dramatic reduction in metabolic activity at only 1 μM (75% loss; Figure 4A). Similar observations were made with 10 μM doses, suggesting that at doses >1 μM there is a “plateau effect,” in that higher doses had no discernible increased effect (Figure 4A). Within the same population of cells, cell death was tested each day by measuring total LDH levels in the media. Results showed significantly high levels of LDH in all CBS‐E2 treatment groups (Figure 4B). The much smaller level of LDH activity in controls was attributed to retention of normal growth rates leading to over‐confluence. Next, CBS‐E2's ability to induce cell death in a non‐cervical cancer human microvascular endothelial cell line (HMEC) was tested. The same LDH leakage assay was performed as above using the highest dose group of TAT‐CaM and CBS‐E2 (10 μM) in both SiHa and HMEC cell lines. Under these conditions, TAT‐CaM alone had a modest impact (34% cell death in HMECs, 26% cell death in SiHa) though this was statistically indistinguishable from buffer controls (Figure 5A). Treatment of HMEC with either 1 or 10 μM CBS‐E2 resulted in another modest increase in cell death over TAT‐CaM‐alone controls (11% and 13% increase, respectively). In contrast, in SiHas significantly high levels of cell death were observed in both the 1 and 10 μM CBS‐E2 treatment groups (Figure 5A). Microscopic analysis of cells on day 3 qualitatively corroborated these results (Figure 5B–D). Collectively, these data support the hypothesis that direct delivery of E2 protein into living cervical cancer cells can inhibit cellular proliferation or induce cell death and, further, suggest that these differential outcomes may be a function of dose. Further, that CBS‐E2 did not induce cell death in the HMEC cell line support a specificity for HPV^+^ cells.

## 4 DISCUSSION

In this study, we describe our use of the efficient, high‐affinity reversible TAT‐CaM adaptor system for CPP‐mediated delivery of CBS‐E2 to cell interiors that exploits natural extra‐ and intracellular levels of calcium. CBS‐E2 cargos were readily delivered into HPV16^+^ cells and showed evidence of sub‐cellular relocalization during cell division. Over time, CBS‐E2 reduced cellular proliferation rates and metabolic activity as well as induced cell death.

CBS‐E2 showed expected high affinity, calcium‐dependent binding kinetics with TAT‐CaM. The calculated K_D_ for CBS‐E2:TAT‐CaM binding was slightly lower that what has been noted for CaM binding to one of its native substrate, endothelial nitric oxide synthase (NOS3),^29^ however this value was well within the range of constants previously determined for other TAT‐CaM cargo proteins.^22^, ^23^ Plateaus observed in the EDTA dissociation phase are confounding in that complete dissociation ought to result in a plateau of 0 nm shift given that nonspecific binding of analyte to the sensor was near zero. However, similar plateaus have been previously observed with CaM and analytes in BLI and were attributed to partial denaturation of the proteins, perhaps a result of tethering to the sensor.^30^ Another contributor is uncertainty of the value of Y_0_ (Y at the beginning of dissociation) as dissociation is very rapid and the instrument takes a reading only every 1.6 s. For very fast processes, there is also often a discontinuity between the end of one phase, in this case dissociation in Ca^2+^, and the next, dissociation in EDTA. Indeed, the residuals indicate the poorest fit at the outset of dissociation, though they remain within the range of normal for the BLI instrument. Regardless of the idiosyncratic uncertainties inherent in the measurements, the kinetics of the interaction were as expected and suitable for delivery of CBS‐E2 into cells.

TAT‐CaM readily delivered CBS‐E2 constructs into the HPV‐16+ cervical cancer cell line SiHa. Over the course of the cell penetration assays, a distinctive and repeatable pattern of intracellular localization in mitotic cells to regions associated with mitotic spindle fibers was observed as well as co‐localization with beta‐tubulin. While both low‐risk (LR) and high‐risk (HR) E2 proteins can associate with the spindle, the manner of association and resultant distribution pattern of LR versus HR E2s during mitosis differs.^26^, ^28^ High‐risk HPV E2 proteins initially cluster at the asters at the onset of mitosis.^26^ As the cell progresses through mitosis, E2 relocates to the midplane where it associates with the Anaphase Promoting Complex (APC/C)^26^, ^28^, ^29^, ^31^ and remains at the midbody through cytokinesis. In concordance with these results, mitotic cells in cell‐penetration experiments showed a distinctive pattern of CBS‐E2 redistribution as previously noted for HR E2.

In this work, CBS‐E2 readily inhibited cellular proliferation and promoted cell death in HPV^+^ cells similar to that as previously reported in E2 reintroduction studies.^13^, ^14^, ^15^, ^16^, ^17^, ^19^, ^20^ TAT‐CaM‐alone‐treated cells exhibited upwards of 18% cytotoxicity in SiHa cells when starting cell counts were sub‐confluent, however, no toxicity was noted at this dosage with 10‐fold higher starting cell counts. This is a documented phenomenon in cancer studies whereby a direct correlation has been drawn between starting cell densities and drug efficacy.^32^ In other works, TAT has documented measurable cytotoxicity above 10 μM^33^ and our metabolic assays employed herein showed no to low cytotoxicity from TAT‐CaM treatment alone. Collectively, these data support that CBS‐E2 mediated the observed phenotypes post‐delivery.

CBS‐E2 failed to inhibit cellular proliferation or induce cell death in the human HMEC cell line supporting previous work showing E2's effects are attributed to interaction with the viral oncoproteins E6 and E7. The mechanism via which E2 mediates these effects, that is, via direct or indirect interaction with E6 and E7, are still unknown. Desaintes et al. found that senescence and apoptosis could occur within the same cellular population and postulated that these outcomes may be the result of the amount of E2 being made within the cell.^16^ In this work, the ability to directly deliver protein into cells allowed control over dosage. Our results support a dose‐dependent effect model whereby E2 inhibits cellular proliferation at low cell‐to‐peptide and promotes cell death at high cell‐to‐peptide ratios. At lower cell‐to‐peptide ratios we did not readily detect cell death, however, it is possible this may be a limitation of approach and more sensitive assays would detect both cell death and reduced proliferation within these populations. Another possible explanation for E2's differential effects is persistence of harder‐to‐treat cancer stem cells (CSCs) within the population.^34^ CSCs persisters have enhanced metastatic potential and would likely be more resistant to E2's effects. Given that SiHas are a heterogenous population, it is possible that high cell‐to‐peptide ratios are required to effectively target and kill CSC persisters within the population. In this current study, we were unable to attribute differential response to distinct cellular populations, however, our future studies are aimed at testing this hypothesis utilizing 3D spheroids with enhanced CSC sub‐populations.^34^ Correlation between cell subtype and E2's effects may further illuminate E2's efficacy both at different stages in carcinogenesis as well as within a heterogeneous tumor population.

In summary, this study showed that the TAT‐CaM adaptor system effectively delivers CBS‐E2 into cultured cells, inhibiting cellular proliferation and inducing cell death. It may hold therapeutic potential as an innovative alternative to transfection or transduction, avoiding problems associated with gene delivery and conferring several advantages including dose control and non‐toxicity. This study provided proof‐of‐concept that TAT‐CaM could serve as a platform for cellular delivery of bioactive CBS‐E2 in living cells. Bioactivity was determined by a reduction of cell proliferation and induction of cell death, known activities of E2. The next steps will be to demonstrate the general utility of the approach by examining multiple cervical cancer cell lines and assess CBS‐E2 delivery in more advanced tumoroid models. To facilitate this, we are working to engineer a cancer‐specific CPP for targeted delivery of E2 into cervical cancer cells within a mixed tumor environment. This work also lays the foundation for a new approach toward our understanding of the biology of HPV‐mediated cervical cancer and studying the specific and interrelated roles of viral proteins in proliferation, senescence, and cell death.

## AUTHOR CONTRIBUTIONS

**Julia C. LeCher:** Data curation (lead); investigation (lead); methodology (equal); supervision (equal); writing – original draft (equal); writing – review and editing (equal). **Hope L. Didier:** Data curation (equal); investigation (equal). **Robert L. Dickson:** Investigation (supporting). **Lauren R. Slaughter:** Investigation (supporting). **J. Camila Bejarano:** Investigation (supporting). **Steven Ho:** Investigation (supporting). **Scott J. Nowak:** Data curation (supporting); formal analysis (supporting). **Carol A. Chrestensen:** Investigation (supporting); methodology (supporting). **Jonathan L McMurry:** Conceptualization (lead); funding acquisition (lead); investigation (lead); project administration (lead); supervision (lead); writing – original draft (equal); writing – review and editing (equal).

## FUNDING INFORMATION

This work was primarily funded by Public Health Service grant R15 EB028609. This work was also supported by Public Health Service grants R16 GM 145448 and R15 HL 161738 to Scott J. Nowak. Hope L. Didier was supported by a Birla Carbon Fellowship from Kennesaw State University College of Science & Mathematics, Kennesaw, GA. Juana C. Bejarano was supported by a Mentor‐Protegee grant from Kennesaw State University College of Science & Mathematics, Kennesaw, GA.

## CONFLICT OF INTEREST STATEMENT

The authors have stated explicitly that there are no conflicts of interest in connection with this article.

## ETHICS STATEMENT

Not applicable.

## Supporting information


**Supplemental Figure 1: Protein purification of CBS‐E2.** E2 was expressed and purified as described. (A) SDS‐PAGE of purification process; samples run are abbreviated: H – homogenate of harvested cells; L – lysate; CL – clarified lysate; FT – flow‐through; W1, W2 – washes; E1, etc – elution fractions of one column volume each. (B) Anti‐E2 immunoblot; “Un” ‐ uninduced cells; and “In” ‐ induced cells.

**Supplemental Figure 2: Co‐Localization of CBS‐E2 and Tubulin with the Nucleus in SiHa cells.** Cervical cancer cells (SiHa) were incubated with fluorescently labeled CBS‐E2 cargo (red) in the presence of equimolar TAT‐CaM for 1 hr. Cells were counterstained with NucBlue (nuclei; blue) then fixed with ice‐cold 100% methanol for 3 min. Post fixation, cells were probed for beta‐tubulin (primary) and detected with a secondary GFP‐conjugate (green). Images were generated on an inverted Zeiss LSM700 Confocal Microscope with Z‐stack projections. Shown at the top and right of each image are orthogonal projections taken at the depth of the nucleus.

## Data Availability

The data that support the findings of this study are available from the corresponding author upon reasonable request.
