# Supplementary figures and images for: Utilization of a cell‐penetrating peptide‐adaptor for delivery of human papillomavirus protein E2 into cervical cancer cells to arrest cell growth and promote cell death
Source: Cancer Rep (Hoboken). 2023 Mar 28;6(5):e1810. doi: 10.1002/cnr2.1810 (PMC10172171; doi:10.1002/cnr2.1810)

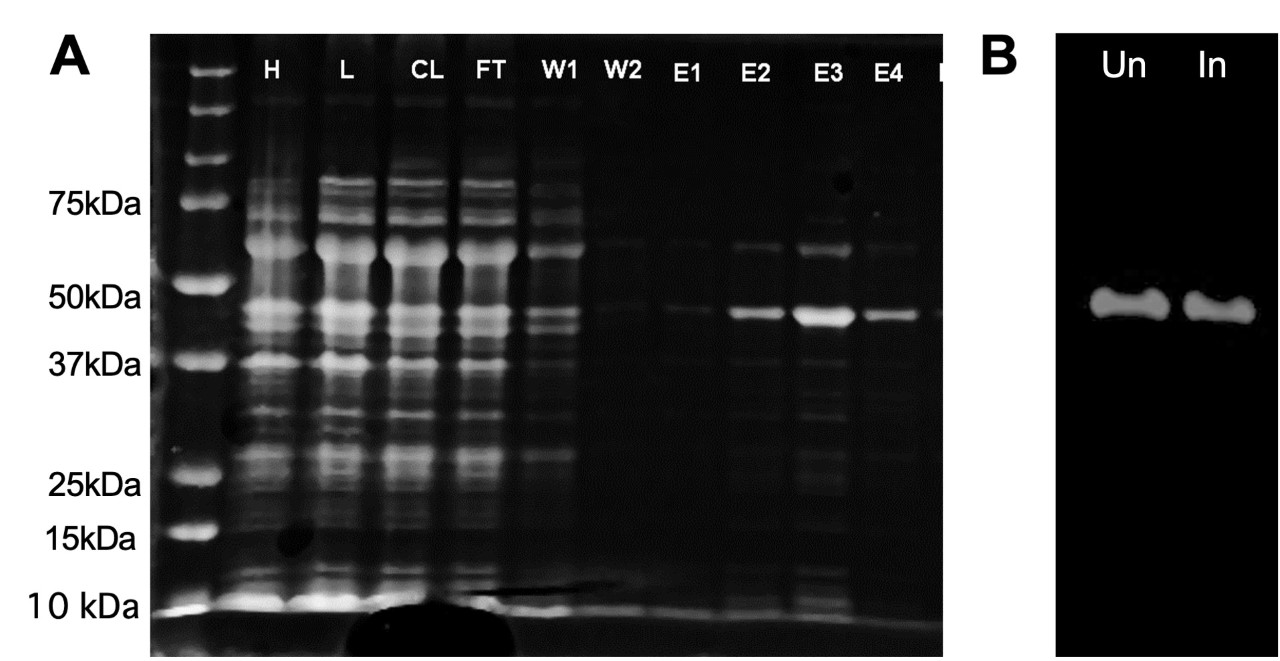

Supplement: Supplementary file 1 — Supplemental Figure 1: Protein purification of CBS‐E2. E2 was expressed and purified as described. (A) SDS‐PAGE of purification process; samples run are abbreviated: H – homogenate of harvested cells; L – lysate; CL – clarified lysate; FT – flow‐through; W1, W2 – washes; E1, etc – elution fractions of one column volume each. (B) Anti‐E2 immunoblot; “Un” ‐ uninduced cells; and “In” ‐ induced cells. [file CNR2-6-e1810-s001.jpg]

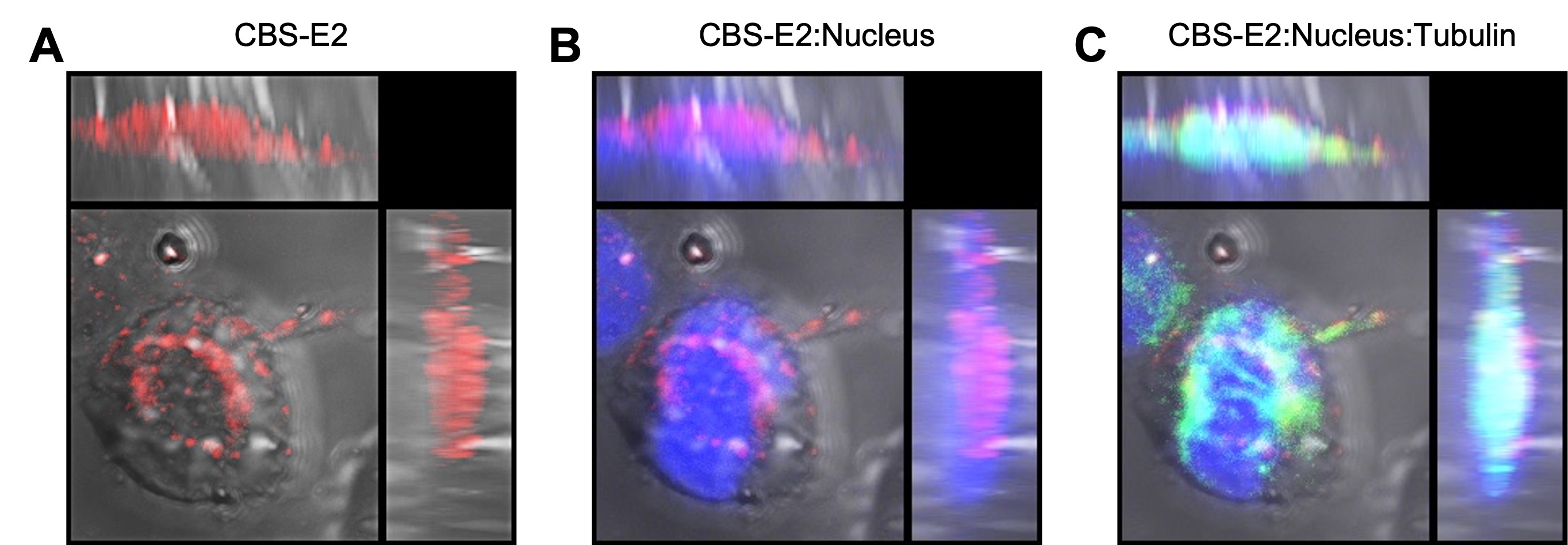

Supplement: Supplementary file 2 — Supplemental Figure 2: Co‐Localization of CBS‐E2 and Tubulin with the Nucleus in SiHa cells. Cervical cancer cells (SiHa) were incubated with fluorescently labeled CBS‐E2 cargo (red) in the presence of equimolar TAT‐CaM for 1 hr. Cells were counterstained with NucBlue (nuclei; blue) then fixed with ice‐cold 100% methanol for 3 min. Post fixation, cells were probed for beta‐tubulin (primary) and detected with a secondary GFP‐conjugate (green). Images were generated on an inverted Zeiss LSM700 Confocal Microscope with Z‐stack projections. Shown at the top and right of each image are orthogonal projections taken at the depth of the nucleus. [file CNR2-6-e1810-s002.jpg]
